# The Implications of Multiple Circadian Clock Origins

**DOI:** 10.1371/journal.pbio.1000062

**Published:** 2009-03-17

**Authors:** Michael Rosbash

## Abstract

Michael Rosbash considers whether the mechanisms that govern circadian rhythms in different organisms have arisen multiple times in evolution, and discusses the implications for our understanding of circadian clocks.

## In the Beginning…

Genetics has had an awesome impact on our understanding of basic processes like circadian rhythms, which were mysterious before the incredibly successful marriage between genetics and recombinant DNA technology about 30 years ago. Subsequent to the pioneering work of Konopka and Benzer [1], genetic screens and DNA sequencing in multiple systems (including but not limited to humans, mice, Drosophila, Neurospora, plants, and cyanobacteria) identified many circadian genes as well as their protein sequences. Coupled with PCR methods to bootstrap from one system to another (e.g., [2,3]), this strategy also revealed that many clock proteins are shared between systems. For example, mammals and Drosophila use orthologs to construct their clocks [4–6]. Fly proteins include the PAS domain-containing transcription factor heterodimer Clock–Cycle (CLK–CYC [orthologs in mammals: CLK–BMAL1]) and the negative regulator Period (PER [orthologs in mammals: PER1, PER2]). These relationships indicate that a similar, basic clock mechanism was present in a common ancestor, before the separation of insects and mammals more than 500 million years ago. Some argue that the relationship of basic clock mechanism and proteins extends to Neurospora [7,8], which would push back the common ancestor date even further. (I am assuming that horizontal gene transfer is not responsible for the commonalities between systems.)

There is, however, no evidence that this relationship extends across the animal–bacterial kingdom divide; i.e., the key circadian proteins of mammals appear completely unrelated to the key circadian proteins of cyanobacteria. Although negative evidence must be interpreted with great caution (“absence of evidence is not evidence of absence”), there is no relationship evident between the circadian proteins of cyanobacteria and those of mammals [4,9]. As this is not the case for many other classes of proteins, the strong suggestion is that circadian rhythms have arisen at least twice, once in an ancestor of present-day cyanobacteria and then again in an ancestor of animals. (More than two evolutionary origins are also possible, as the different set of plant circadian proteins may indicate a third independent origin; although see below.) As some early version of cyanobacteria are generally credited with the rise of oxygen about 2.4 billion years ago, and multicellular eukaryotes did not appear for another 1.5 billion years or so [10], the evolution of cyanobacterial rhythms was probably well before that of eukaryotic rhythms.

## The Importance of Biochemistry

A multiple-origin view of circadian clock origins has implications for how animal clocks keep time; i.e., what are their mechanisms or “quartz crystals”? In other words, progress in one system may have no impact on understanding a second. Relevant also is the fact that genetics is a poor way to define mechanism, in contrast to its irrefutable value in identifying key genes and proteins. These sequences are seductive, as a link between a circadian gene and a transcription factor can be interpreted to indicate an intimate relationship between transcription and timekeeping (e.g., [11]). However, only biochemistry can rigorously define mechanism, and nowhere is the distinction with genetics better illustrated than in the breathtaking reconstruction of a cyanobacterial clock in vitro [12].

In vivo genetics and physiology suggested that the Kai system of cyanobacteria functions to regulate transcription and that a transcriptional feedback loop is therefore the key to mechanism [13]. This paradigm came from the animal clock world, because it was first shown in flies that mRNA levels of a key clock undergo oscillations due to transcriptional feedback [14,15]. However, subsequent work by Kondo and colleagues, especially their in vitro biochemistry, indicates that a post-translational phosphorylation–dephosphorylation cycle of the Kai A-B-C complex is the quartz crystal of cyanobacteria, with transcriptional rhythms a largely downstream consequence or of secondary value [12,16]. Important work on this system has continued [17,18], and recent in vitro comparisons of mutant KaiC proteins suggest that the KaiC ATPase or phosphatase activity is the key timekeeper for the entire cyanobacterial 24-hour cycle [19]. If this is the case, then a single overarching enzyme and mechanism may be rate-limiting for this circadian cycle, consistent with a limit cycle mechanism. This description is almost certainly an oversimplification, as conformational states or aggregates of states are also likely to be important for mechanism [20,21]. In addition, very recent data complicate this highly circumscribed view and suggest that transcription is not as irrelevant as previously believed, or that it may constitute a second circadian oscillator that ticks away in parallel within each bacterium [22]. Although the importance of these new findings is not yet clear, the key point is that in vitro biochemistry has been indispensible for understanding the cyanobacterial clock mechanism.

## Kinases and Animal Rhythms

In contrast to cyanobacteria, eukaryotic circadian systems are notably lacking in comparable biochemistry that addresses timekeeping mechanisms. (To keep matters circumscribed, I will focus on animals and especially Drosophila.) Given the lack of evidence for common proteins and a common ancestor of animals and cyanobacterial circadian systems, it is unlikely that they have a mechanistic relationship. If indeed truly of independent origin, the two systems could still reflect convergent evolution, e.g., hitting upon the same mechanism by accident or because it is the only way to keep circadian time. It is more likely, however, that the cyanobacterial mechanism is misleading for understanding animal clock mechanisms.

Nonetheless, this work has had an enormous influence on the animal clock world. This is because the KaiC phosphorylation cycle makes a seductive link to the phosphorylation events that animal clock transcription factors experience [23–26]. In other words, these proteins not only function in circadian transcriptional feedback but are also subject to temporally regulated phosphorylation. Moreover, the same clock kinases have been identified in both Drosophila and mammalian systems [6,27–29], suggesting that this feature of circadian rhythms was already present in a common ancestor of flies and mammals. To extend the analogy with cyanobacteria, many now believe that this post-translational regulation is more central to timekeeping than the initially proposed and embraced transcriptional feedback loop. For example, dramatic period length alterations are more frequently found in mutants that affect post-translational regulation (e.g., [26,30]), and there is evidence that aspects of transcriptional feedback are not necessary for circadian rhythmicity [31]. A real test requires disabling the ability of a key positive transcription factor like CLK to experience transcriptional feedback in an animal (e.g., [32]). Behavioral rhythmicity would then make a strong case for the primacy of post-translational regulation.

## Genetics and Rate-Limiting Steps

Given the absence of relevant in vitro experiments, what can genetics say about mechanism or the rate-limiting timing steps of animal circadian clocks? First, it is important to emphasize that mutant or overexpression studies, even in vivo, do not define rate-limiting steps for a wild-type clock. Although a mutant enzyme is likely to become rate-limiting for timekeeping of the mutant clock, there is no reason why the same activity should be rate-limiting in the context of a wild-type clock. Even if the mutant effect on K_m_ or V_max_ can be measured in vitro and is modest, this has not been done in vivo (admittedly difficult) nor in vitro in the context of bona fide circadian timekeeping as in the cyanobacterial experiments. Given that the in vivo effects are therefore unknown, it seems possible that many mutations with period effects move their in vivo enzyme activities far from their normal K_m_ or V_max_ values. The same argument applies to small molecule screens. Kinases are known to be good drug targets (transcription factors, in contrast, are poor drug targets), and it therefore makes sense that small molecule inhibitors used in period screens will successfully target kinases and will decrease an activity sufficiently to make it rate-limiting [33]. However, Occam's razor suggests that the activities of conserved circadian kinases like casein kinase I e (CKIe [*doubletime* in flies; *tau* in mammals]) are unlikely to be rate-limiting for period determination in fully wild-type circumstances—despite being critically involved in timekeeping.

A perhaps broader view is that post-translational regulation, the rate of PER turnover in flies as an example, may be partly rate-limiting for period determination, and DBT (Doubletime) activity on PER is one aspect of PER turnover biochemistry. However, even this is uncertain, and it is certainly unknown which protein turnover steps, e.g., *period* protein phosphorylation and/or recognition of the protein by its E3 ligase Slimb [26], are actually rate-determining in the wild-type context.

## Increasing Gene Dose and Transcription

There are, however, a few interesting and simple genetic approaches of mechanistic value, including dosage sensitivity. Which genes substantially affect circadian period with a simple 2× change in gene dose? The logical assumption is that a 2× change in gene dose will have a modest, approximately 2-fold effect on gene activity and protein level. A consequent period effect may therefore indicate a rate-limiting step. Konopka originally appreciated this point and used classical Drosophila genetic approaches to show that an increase in *period* gene dose shortened period length [34]. This observation was subsequently confirmed by others using transgenic technology [35,36]. More recently, a modest decrease in *vrille* (*vri*) gene dose (VRI is another transcription factor clock gene) decreased circadian period [37], and a modest increase in *Clock* gene dose also decreased period length [38]. An increase in the dose of the orthologous *Clock* gene in mammals had a similar effect in that system [39]. Interestingly, all three of these genes encode transcription factors, and it is notable that there is no comparable (modest) gene dose–period effect reported for clock kinases. This might indicate that it is the transcription of clock genes that is rate-limiting for circadian period. On the other hand, an increase in *period* gene dose might have a more primary effect on the rate of *period* protein phosphorylation and only a secondary effect on transcription; a similar possibility applies to alterations in *vri* and *Clk* gene dose.

Relevant to this pro-transcription argument is our recent demonstration that adding the strong transcriptional activator viral protein 16 (VP16) to the positive transcription factor CYC strongly increases clock gene transcription and decreases period length [38]. On the other hand, the substantial transcription effects of CYC–VP16 make the result subject to the same criticism applied above to kinases. Perhaps a better pro-transcription argument is that the promoter of the direct CLK–CYC target gene is necessary for the short period characteristic of the CYC–VP16 strain. In addition, increases in *period* gene dose with a heterologous promoter lengthen rather than shorten circadian period [38]. This suggests that the effects of increasing CLK levels might shorten period by predominantly increasing the rate of *period* transcription.

## Multiple Rate-Limiting Steps?

**Figure 1:**
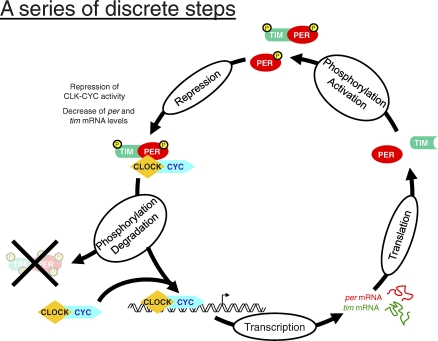
A Schematic Picture of the Drosophila Circadian Cycle as a Series of Discrete Steps An oversimplified view that depicts each step in the cycle (transcription, translation, etc.) as being temporally and mechanistically separated from all other steps.

It is important to emphasize that a transcription-centric view of circadian period control is not incompatible with a parallel post-translational-centric view, emphasizing for example protein turnover. One possibility is that both processes contribute to a more robust oscillator [40]. Another nonexclusive possibility is that both transcriptional and post-transcriptional regulation control period but at different phases of the circadian cycle. This alternative proposal of multiple rate-limiting steps emphasizes a cell cycle-like circadian program, in which different phases of the cycle occur in sequence, like G1-S-G2-M, with different rate-limiting steps at different phases of the cycle. (This is an analogy, not a proposal that rate-limiting steps that govern the circadian cycle are related to or even similar to those that govern the cell cycle.) For example, PER protein turnover might be rate-limiting at one phase of the cycle. Degradation of this transcriptional repressor below some threshold value would then be required for transcription to recommence and become a subsequent and very different rate-limiting step (Figure 1). It is noteworthy that this almost certainly oversimplified view is not dramatically different from what was suggested based on the original *per* RNA, transcription, and *per* protein western blot observations about 15 years ago [14,15,23].

This view is, however, dramatically different from the current, single rate-limiting process that dominates the cyanobacterial circadian world: although armed with multiple subroutines, a single enzyme activity is probably rate-limiting for the entire 24-hour cycle [19]. Importantly, a common ancestor or a single origin for both animal and cyanobacterial clocks would make this multiple rate-limiting step proposal much less likely.

## Temperature Compensation

How to achieve the enigmatic process of temperature compensation is a troubling but unavoidable complication of a multiple rate-limiting step view of animal circadian clocks. Temperature compensation reflects the fact that circadian period length changes little as a function of temperature (within a physiological range; Q_10_ ~1.0). For multiple rate-limiting steps, there must then be multiple mechanisms of temperature compensation, at least one for each rate-limiting reaction. Balancing kinases and phosphatases that govern each step and have comparable temperature coefficients is a single mechanism that could apply to different kinase–phosphatase pairs acting on different substrates at different cycle times. There are also other solutions, including an overall measuring device that keeps the cycle at 24 hours and a balanced expansion–contraction of different steps. The latter should be easy to disprove, by examining for example the relative length of the transcription and the protein turnover phases of the circadian cycle at different temperatures. Temperature compensation may even apply more broadly to other physiological processes; i.e., circadian rhythms may not be so special from this point of view. In any case, temperature compensation of the cyanobacterial circadian system is likely to have a single solution, which may be different from the eukaryotic solution(s) and reflect the independent origins of the two systems.

## Transcription and the Origins of Circadian Clocks

Finally, what were the original selective advantages, the driving forces, for the origins/development of rhythms in the eukaryotic and bacterial systems? Present-day differences between prokaryotic and eukaryotic circadian systems in their relationship to transcription, DNA, and light offer possible clues.

Circadian rhythms are important for the fitness of a photosynthetic organism like cyanobacteria [41,42]. Many cyanobacterial species also organize a large fraction of their metabolism under circadian transcriptional control (e.g., [43]), implying for example a selective advantage to transcribing photosynthesis genes at the right time during a light–dark cycle. Moreover, in species that do not spatially segregate nitrogen fixation and photosynthesis, oxygen sensitivity of the former demands temporal segregation from the latter [44]. So although circadian transcription may not be essential for some cyanobacterial timekeeping features, its temporal organization may have provided a progenitor with a sufficient selective advantage to drive the development of rhythms. (A similar point has been made by McKnight and colleagues based on the temporal organization of yeast metabolism, including the segregation of reductive and oxidative functions [45,46].) This bottom-up view suggests that the Kai transcription factors of a photosynthetic progenitor of current-day cyanobacteria developed the capacity to orchestrate transcription in response to the ever-present light–dark cycle, and eventually to anticipate that cycle in a transcription- and even light-independent manner.

Eukaryotes, in contrast, show rather little pan-tissue circadian transcription—with the notable exception of central clock genes [47]. Although other interpretations are possible, this suggests that circadian output transcription, critical to many tissue-specific functions including metabolism, did not drive the development of eukaryotic rhythms but developed secondarily. This is despite the intimate relationship between circadian transcription and metabolism in certain tissues of present-day mammals, e.g., [48,49].

## Light and the Origins of Circadian Clocks

My view of eukaryotic clock origins was inspired by the fact that cryptochrome is a circadian light-sensor in insects [50,51]. As cryptochrome has a close relationship to photolyase, an enzyme that uses light to repair UV-induced DNA damage [52], responding to light-induced DNA damage may have been a major driving force for the development of circadian rhythms in eukaryotes [51,53–55]. In this context, it is intriguing that DNA synthesis, DNA repair, and the cell cycle have been recently shown to have a closer relationship with circadian rhythms and light than previously imagined, in Neurospora as well as metazoans [46,55,56]. For example, the S phase in zebrafish is under light and circadian control [57], and mammalian homologs of the Drosophila clock protein Timeless (TIM) function in the DNA damage response and perhaps also the cell cycle [58–60]. Given the important role played by signal transduction in DNA repair, the relationship of DNA damage and repair to rhythms may have additional explanatory power, namely, the origin of circadian kinases. Intriguingly, at least one enzyme is important for plant as well as animal and Neurospora clocks and also for other major signal transduction systems, e.g., CKII [6,61,62]. In other words, the regular, daily appearance of light as well as the consequent regular induction of DNA damage and repair systems may have provided the molecular tool kit (photolyase, a TIM ancestor, and even kinases) for the development of circadian rhythmicity. This largely negative relationship between light and eukaryotic circadian rhythms, “flight from light” [53], further underscores the contrast with photosynthetic cyanobacteria.

## Abbreviations

CK: casein kinase
*clk*: *clock*
*cyc*: *cycle*
*per*: *period*
VP16: viral protein 16
*vri*: *vrille*
